# Impact of Interfractional Error on Dosiomic Features

**DOI:** 10.3389/fonc.2022.726896

**Published:** 2022-06-10

**Authors:** Chanon Puttanawarut, Nat Sirirutbunkajorn, Narisara Tawong, Suphalak Khachonkham, Poompis Pattaranutaporn, Yodchanan Wongsawat

**Affiliations:** ^1^ Chakri Naruebodindra Medical Institute, Ramathibodi Hospital, Mahidol University, Samutprakarn, Thailand; ^2^ Brain-Computer Interface Laboratory, Department of Biomedical Engineering, Faculty of Engineering, Mahidol University, Nakhorn Pathom, Thailand; ^3^ Department of Diagnostic and Therapeutic Radiology, Ramathibodi Hospital, Mahidol University, Bangkok, Thailand

**Keywords:** dosiomics, radiomics, texture analysis, dose distribution, stability, generalizability, interfractional error

## Abstract

**Objectives:**

The purpose of this study was to investigate the stability of dosiomic features under random interfractional error. We investigated the differences in the values of features with different fractions and the error in the values of dosiomic features under interfractional error.

**Material and Methods:**

The isocenters of the treatment plans of 15 lung cancer patients were translated by a maximum of ±3 mm in each axis with a mean of (0, 0, 0) and a standard deviation of (1.2, 1.2, 1.2) mm in the x, y, and z directions for each fraction. A total of 81 dose distributions for each patient were then calculated considering four fraction number groups (2, 10, 20, and 30). A total of 93 dosiomic features were extracted from each dose distribution in four different regions of interest (ROIs): gross tumor volume (GTV), planning target volume (PTV), heart, and both lungs. The stability of dosiomic features was analyzed for each fraction number group by the coefficient of variation (CV) and intraclass correlation coefficient (ICC). The agreements in the means of dosiomic features among the four fraction number groups were tested by ICC. The percent differences (PD) between the dosiomic features extracted from the original dose distribution and the dosiomic features extracted from the dose distribution with interfractional error were calculated.

**Results:**

Eleven out of 93 dosiomic features demonstrated a large CV (CV ≥ 20%). Overall CV values were highest in GTV ROIs and lowest in lung ROIs. The stability of dosiomic features decreased as the total number of fractions decreased. The ICC results showed that five out of 93 dosiomic features had an ICC lower than 0.75, which indicates intermediate or poor stability under interfractional error. The mean dosiomic feature values were shown to be consistent with different numbers of fractions (ICC ≥ 0.9). Some of the dosiomic features had PD greater than 50% and showed different PD values with different numbers of fractions.

**Conclusion:**

Some dosiomic features have low stability under interfractional error. The stability and values of the dosiomic features were affected by the total number of fractions. The effect of interfractional error on dosiomic features should be considered in further studies regarding dosiomics for reproducible results.

## Introduction

In radiation therapy, radiation dose information is analyzed to determine an appropriate radiation plan. Dosimetric values derived from organ-at-risk dose-volume histograms (DVHs) or dosimetric features, such as the mean dose or V20, are commonly used to estimate the normal tissue complication probability (NTCP). However, dosimetric features do not incorporate spatial information from the dose distribution. Therefore, texture analysis of dose distributions, called dosiomics, has been proposed (1–3). Studies have shown that dosiomics can be used to predict complications from radiation therapy more accurately than dosimetric features (1, 3), yet there exist some concerns regarding the stability and generalizability of texture analysis (4, 5). Some dosiomic features were found to be unstable over different grid resolutions (6), dose calculation algorithms (6), and cube pixel spacing (7). This shows that the reproducibility of dosiomic features depends on the process of producing images.

Geometric errors in radiotherapy can be from random and systematic errors. Systematic geometrical error results in a total shift of the dose distribution, while random geometrical error, defined as interfractional error in this study, leads to blurring of the dose distribution (8). During treatment delivery, many random errors, such as setup error, organ shift, and respiratory motion can result in dose deviation from the original plan. Therefore, the actual dose distribution the patient receives could differ from the original treatment plan. These errors can also result in variations in the dose distribution within the same patient and the same treatment plan. The impact of setup errors in dosimetric features has been reported (9–12). Furthermore, these errors can result in overestimation or underestimation of probability according to the NTCP model (13–15). In other words, these errors might decrease the reproducibility of dosiomics as well.

For many cancers, the radiation dose and the number of fractions can vary from patient to patient. Because of interfractional error, a different total number of fractions may induce different error behavior. Random errors in the interfractional dose distribution cause the dose distribution to appear more blurred for a larger total number of fractions than for a smaller total number of fractions. These errors may also further affect the reproducibility of dosiomic features. To study the effect of such errors on dosiomic features, we investigated the following:

- The stability of dosiomic features extracted from dose distributions with interfractional error.
- The differences in dosiomic features extracted from original dose distributions and dose distributions with interfractional error.

## Materials and Methods

### Datasets

Original CT image data, original dose distributions, treatment plan data, and regions of interest (ROIs) for 15 lung cancer patients from the Varian Eclipse Treatment Planning System (TPS; version 16.1, Varian Medical Systems, Palo Alto, CA, USA) at Ramathibodi Hospital were used in this study. Four different ROIs, including the GTV, PTV, heart, and both lungs, were labeled by radiation oncologists. All of the treatment plans corresponded to IMRT/VMAT for lung cancer. The information for clinical factors, radiation setting, and some dose profiles of all patients are included in **Supplementary Tables S1-S3**.

### Error Simulation

We simulated the interfractional error by introducing Gaussian error into each fraction. Gaussian error was selected because the sum of arbitrary errors can be approximated as Gaussian error by the central limit theorem (16). The interfractional errors were simulated in Varian Eclipse Treatment Planning System by shifting the isocenter of the original treatment plans n times, where n is the number of fractions. The mean error was (0, 0, 0), and the standard deviation was (1.2, 1.2, 1.2) mm for the Gaussian distribution in the x, y, and z directions (with a maximum shift of ± 3.0 mm in the x, y, and z directions). The range of error -3.0 mm to 3.0 mm is used because in our institution, error < 3 mm is mostly acceptable and still within the PTV margin for IMRT/VMAT treatment. Therefore, for errors > 3 mm, a correction was applied, and the effect of error in that scenario did not exceed 3 mm.

The shifted treatment plans were used to calculate the dose distribution and the accumulative n dose to yield the final dose distribution (D_err_). The dose distribution of the plan without errors (D_ori_) was also recalculated to ensure that the parameters of the dose calculation were the same. To generate more dose distributions, we randomly sampled error from a Gaussian distribution to simulate 20 samples of D_err_ for each patient (with the same interfractional error for the same total number of fractions for different patients) and varied the total number of fractions among 2, 10, 20, and 30 while keeping the same total dose.

**Figure 1:**
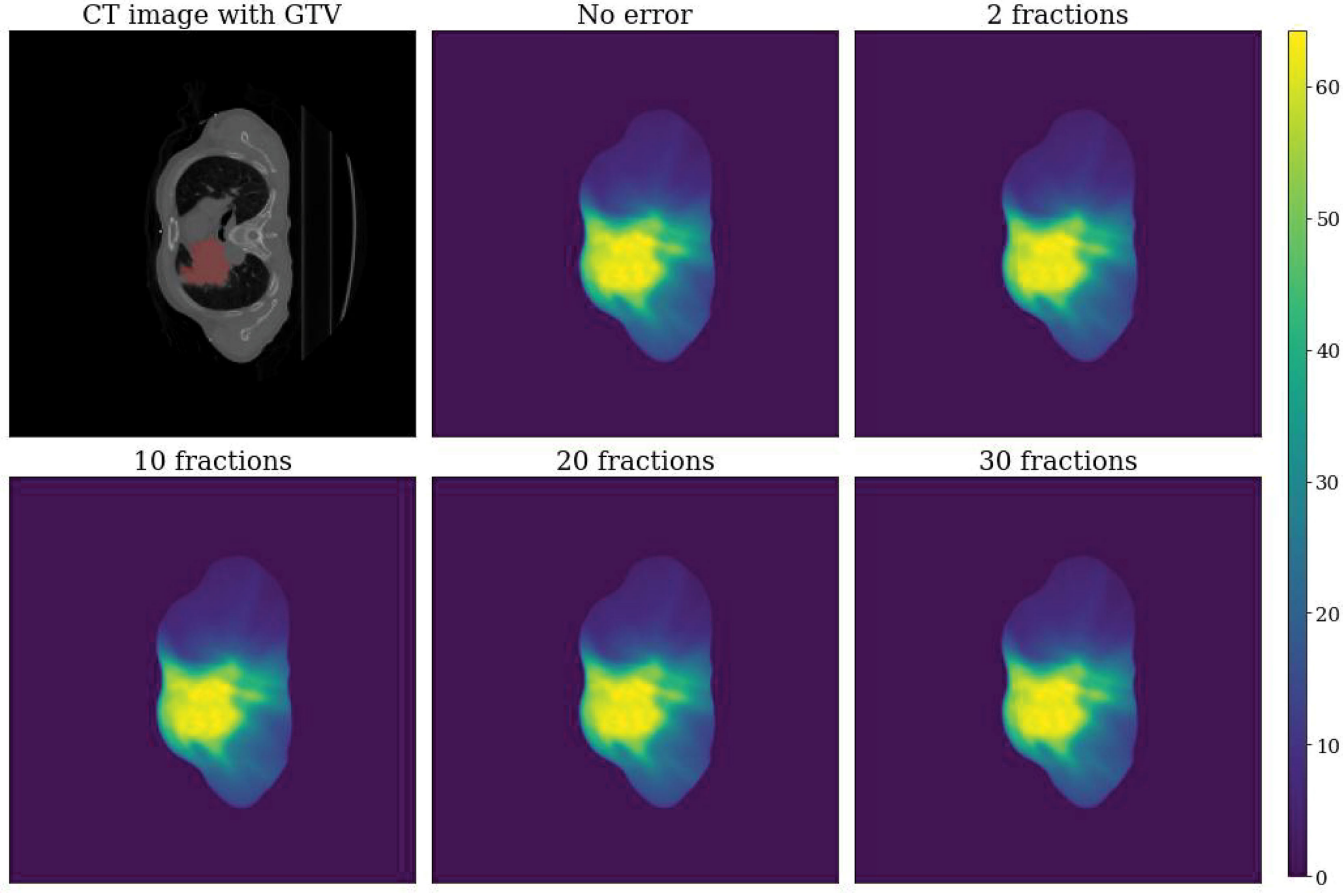
Example dose distributions for patient 9. Top left: CT image with GTV label. Top center: Dose distribution without error (D_ori_). Top right: Dose distribution with error for a total of two fractions. Bottom left: Dose distribution with error for a total of 10 fractions. Bottom center: Dose distribution with error for a total of 20 fractions. Bottom right: Dose distribution with error for a total of 30 fractions.

**Figure 2:**
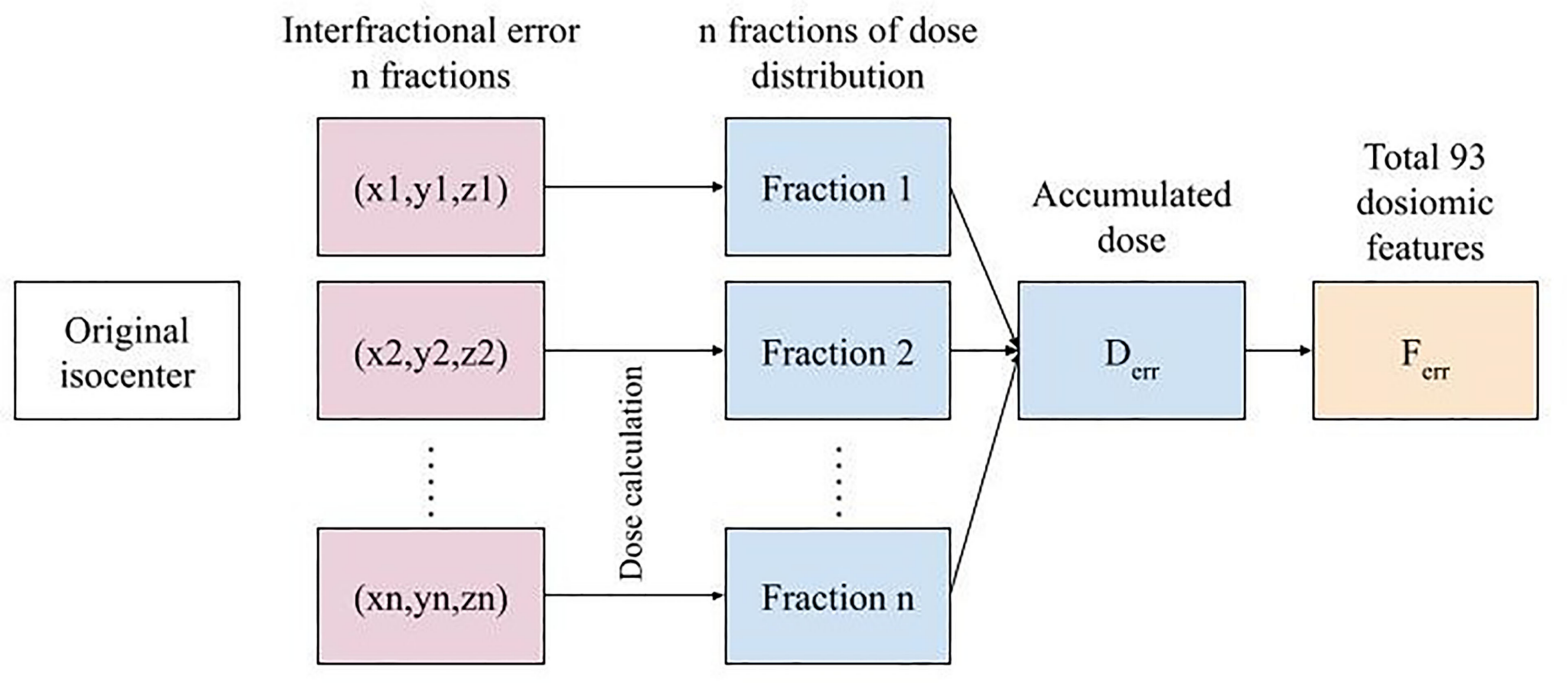
The overall error simulation procedure for n fractions to feature extraction for a single treatment plan.

An example of a simulated dose distribution for patient 9 is shown in **Figure 1**. The overall process from error simulation to feature extraction is shown in **Figure 2**. The dose calculations for D_ori_ and D_err_ were performed using the analytical anisotropic algorithm (AAA) on Varian Eclipse™ TPS. The dose calculation grid resolution was set to 2.5 cm^3^. A total of 81 dose distributions (20 images for each fraction group and one nonshifted dose distribution) were calculated for each patient.

### Features

**Figure 3:**
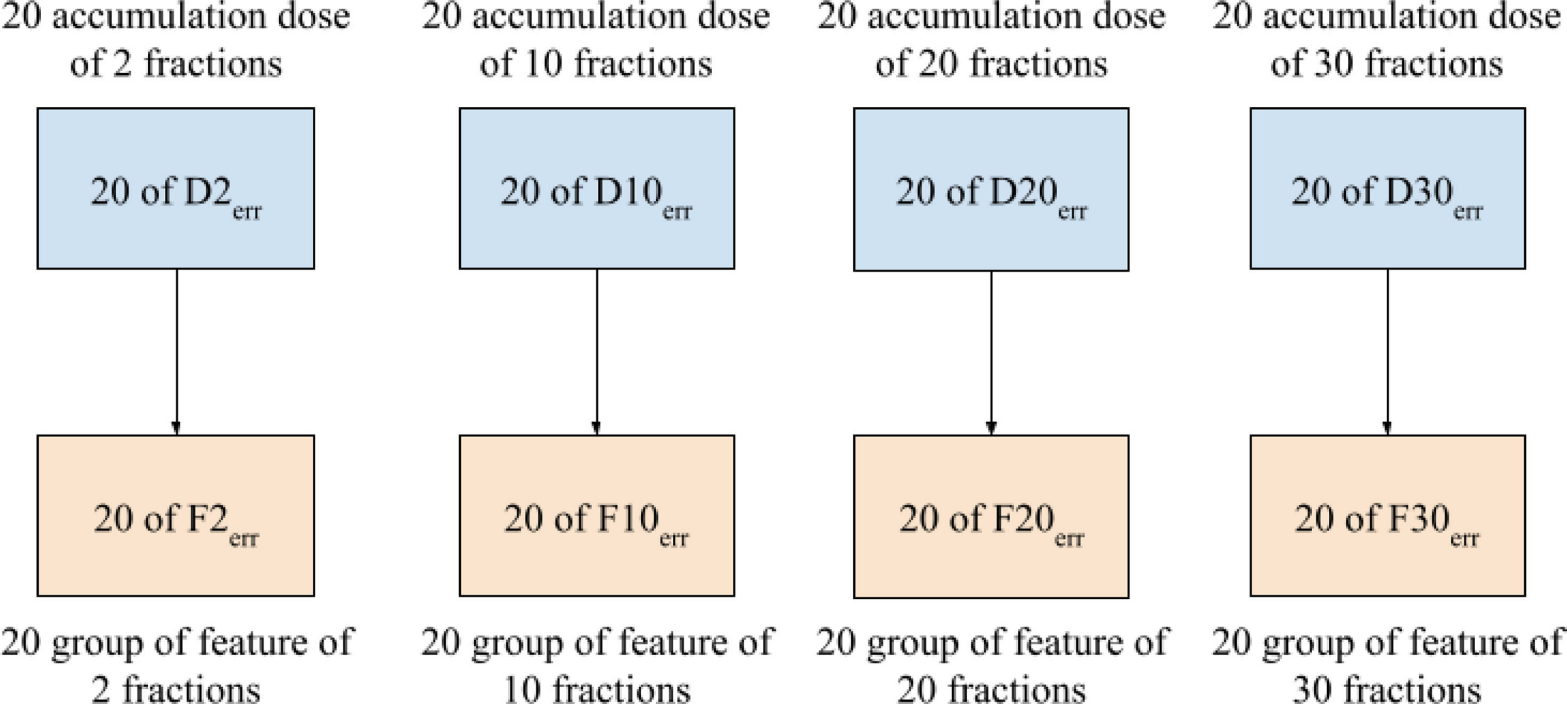
Total of 80 groups of features extracted from D_err_ for each patient.

Dosiomic features were extracted from the dose distributions by using the pyradiomics library, an open-source Python package for extracting radiomic features from medical images (17). The features considered in this study were in compliance with the feature definitions described by the Imaging Biomarker Standardization Initiative (IBSI) (18). Before calculating a feature, dose distribution data were binned into 70 discrete levels from 0-70 Gy. Dosiomic feature extraction was performed within the ROIs of the GTV, PTV, heart, and both lungs (bilateral lungs subtracted by GTV), as labeled by radiation oncologists. Features were extracted from D_err_ to obtain sets of dosiomic features for the total number of fractions corresponding to 2, 10, 20, and 30, denoted as F2_err_, F10_err_, F20_err,_ and F30_err_, respectively, as shown in **Figure 3**. We also extracted features from D_ori_ to obtain F_ori_. We denoted FX_err_ as dosiomic features calculated from DX_err_ for any X total number of fractions. A total of 93 dosiomic features were calculated in this study: first-order features (18 features), gray-level cooccurrence matrix (GLCM) (24 features), gray-level run-length matrix (GLRLM) (16 features), gray-level size zone matrix (GLSZM) (16 features), neighboring gray-tone difference matrix (NGTDM) (five features), and gray-level dependence matrix (GLDM) (14 features). For a list of all features included in the study, we refer to **Supplementary Table S4**.

### Data Analysis

Stability analysis of dosiomic features under interfractional errors was performed by the coefficient of variation (CV) and intraclass correlation coefficient (ICC) for each feature *f_err_* ∈ F_err_ over the 20 accumulated dose distributions for the different total numbers of fractions, as follows:


$$CV=\left|\frac{{\text{σ}}_{f}}{f_{avg}}\right|\times 100\%$$

for *f_avg_* is the mean, and *σ_f_* is the standard deviation of the dosiomic features *f_err_*. *f_avg_* and *σ_f_* were calculated by $f_{avg}=\frac{\sum_{i=1}^{20}f_{err,i}}{20}$ and $\sigma_{f}=\frac{\sqrt{\sum_{i=1}^{20}{\left(f_{err,i}-f_{avg}\right)}^{2}}}{19}$ , respectively. CV has been used in previous studies for radiomic feature stability analysis (19–22). The CV was calculated for all features and all total numbers of fraction groups. The CVs of all features were categorized into four groups as follows: very small CV (CV < 5%), small CV (5% ≤ CV < 10%), intermediate CV (10% ≤ CV < 20%), and large CV (CV ≥ 20%). The CV results are reported as the average CV among all patients. Another stability test under interfractional errors was performed by ICC (6, 23, 24) of a single-measurement, absolute-agreement, two-way random effect model. ICC is a statistical measure of agreement between different raters; in this case, different interfractional errors give different dosiomic values to the subjects (25). A threshold of ICC ≥ 0.9 indicated good stability under interfractional error (26, 27).

The differences among the values of features extracted from the original dose distribution (F_ori_) and the features extracted from the dose distribution with interfractional error (F_err_) were calculated according to the percent difference (PD) over all patients. PD was calculated by


$$PD=\sum_{i=1}^{20}\frac{f_{err,i}-f_{ori}}{f_{ori}}\times \frac{1}{20}\times 100\%$$

where *f_err_* ∈ F_er_ and *f_ori_* ∈ F_ori_. PDs were calculated for all features and all total numbers of fraction groups. The PD results were reported as the average PD among all patients.The effect of the total number of fractions on the mean dosiomic features was analyzed for all patients by ICC by comparing the features of each patient that were extracted from four groups corresponding to different total numbers of fractions.

All statistical analyses were performed by using in-house software implemented in the Python programming language. A p-value less than 0.01 indicated that the test was significant.

## Results

**Figure 4:**
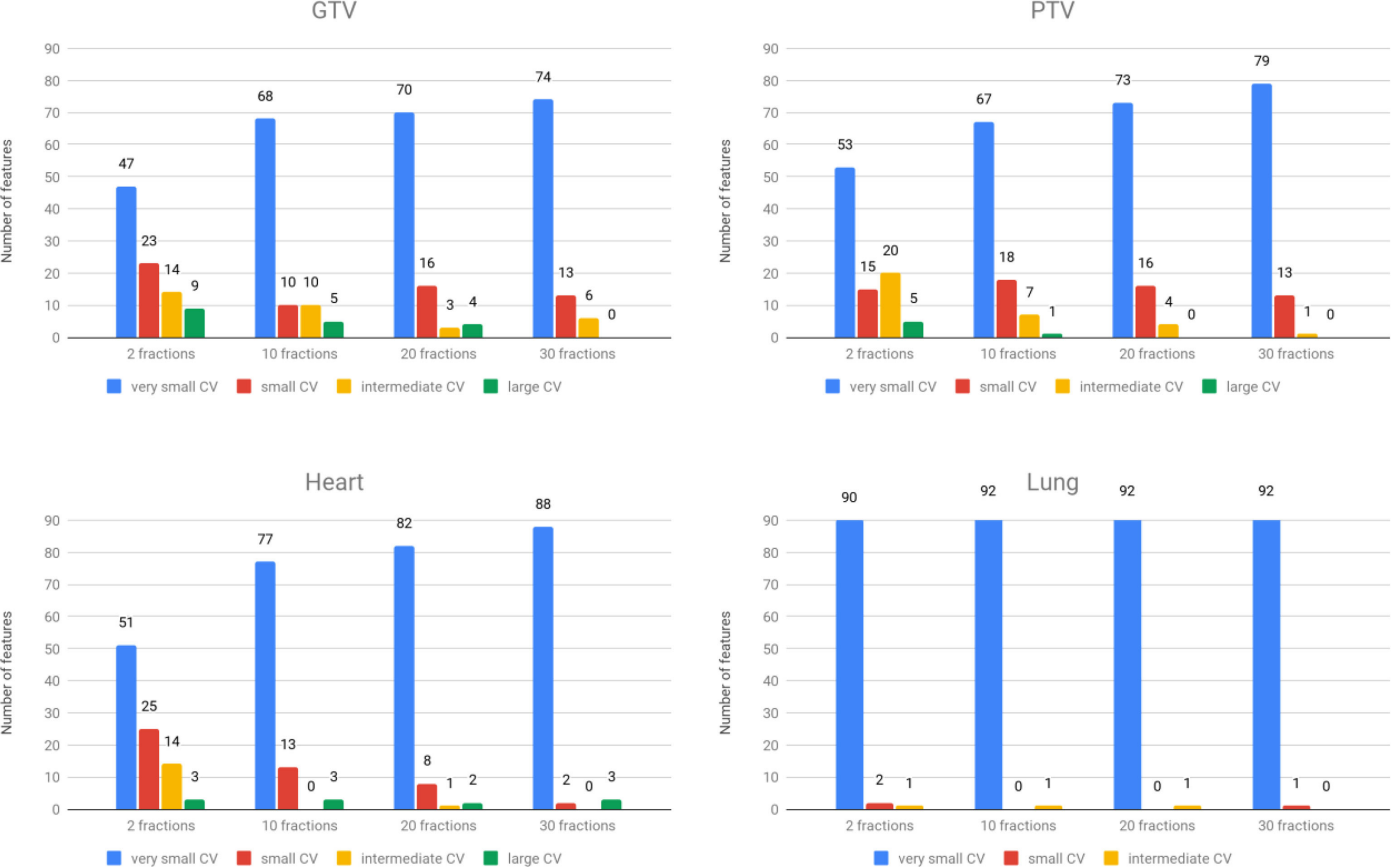
The number of features was categorized into four groups based on CVs. Top left: GTV region. Top right: PTV region. Bottom left: heart region. Bottom right: lung region.

Stability under interfractional error was analyzed for each group and ROI. A summary of CV is shown in **Figure 4**. The results show that the overall stability of dosiomic features decreased with decreasing total number of fractions. When comparing four different ROIs, GTV and PTV had more features with large CVs than the other groups. Among the five groups, the GLSZM features were less stable under interfractional error than other feature classes (**Supplementary Figure S1**). Most of the features in the “lung” region had CV less than 10%, except “small area low gray-level emphasis” from GLSZM in Groups F2_err_, F10_err,_ and F20_err_ (**Supplementary Figure S1**). Dosiomic features with large CVs (CV ≥ 20%) were “Skewness” (2 times) and “minimum” (1 time) from first-order statistics, “ClusterShade” (4 times) and “ClusterProminence” (1 time) from GLCM, “SizeZoneNonUniformity” (3 times), “SizeZoneNonUniformityNormalized” (1 time), “SmallAreaEmphasis” (5 times), “SmallAreaHighGrayLevelEmphasis” (7 times), and “SmallAreaLowGrayLevelEmphasis” (7 times) from GLSZM, and “Complexity” (2 times) and “Strength” (2 times) from NGTDM (**Supplementary Figure S1**). There were no features that had a CV greater than 50%. Additional details of CV regarding each of the dosiomic features in all groups are provided in **Supplementary Figure S1**.

From the ICC results, some dosiomic features were found to have an ICC < 0.9 (p-value < 0.01) (**Table 1**). From across four different ROIs, GTV and PTV had the highest number of features, with ICC < 0.9, and lung was the lowest. The result from ICC was also similar to CV, which showed that there were more features with ICC < 0.9 when decreasing the number of fractions. The overall ICC results showed that the GLSZM dosiomic features had a lower ICC than the other groups. The common features in all groups that had ICC < 0.9 were “SmallAreaLowGrayLevelEmphasis” and “SmallAreaHighGrayLevelEmphasis” from GLSZM. The features that had ICC < 0.9 are shown in **Table 1**. The value of 1 - ICC for all features is shown in **Supplementary Figure S3**.

**Table 1:** Features that had ICC < 0.9 are shown for different ROIs and different numbers of fractions.

Number of fractions (n)	(0.75 ≤ ICC < 0.9)	(0.5 ≤ ICC < 0.75)	(ICC < 0.5)
**GTV 2 fractions (8)**	**GLSZM** Gray Level Non Uniformity Normalized **NGTDM** Contrast **GLDM** Dependence Non Uniformity Normalized Dependence Variance	**GLSZM** Size Zone Non Uniformity Normalized Small Area Emphasis Small Area High Gray Level Emphasis Small Area Low Gray Level Emphasis	
**GTV 10 fractions (4)**	**GLSZM** Small Area High Gray Level Emphasis	**GLSZM** Size Zone Non Uniformity Normalized Small Area Emphasis Small Area Low Gray Level Emphasis	
**GTV 20 fractions (4)**	**GLSZM** Small Area Emphasis Small Area High Gray Level Emphasis Small Area Low Gray Level Emphasis	**GLSZM** Size Zone Non Uniformity Normalized	
**GTV 30 fractions (4)**	**GLSZM** Size Zone Non Uniformity Normalized Small Area Emphasis Small Area High Gray Level Emphasis Small Area Low Gray Level Emphasis		
**PTV 2 fractions (4)**	**GLCM** Cluster Shade **GLSZM** Small Area Emphasis	**GLCM** Cluster Prominence **GLSZM** Small Area High Gray Level Emphasis	
**PTV 10 fractions (1)**	**GLCM** Small Area High Gray Level Emphasis		
**PTV 20 fractions (1)**	**GLSZM** Small Area High Gray Level Emphasis		
**PTV 30 fractions (1)**	**GLSZM** Small Area High Gray Level Emphasis		
**Heart 2 fractions (2)**	**GLSZM** Size Zone Non Uniformity Normalized		**GLSZM** Small Area Low Gray Level Emphasis
**Heart 10 fractions (1)**			**GLSZM** Small Area Low Gray Level Emphasis
**Heart 20 fractions (1)**			**GLSZM** Small Area Low Gray Level Emphasis
**Heart 30 fractions (1)**			**GLSZM** Small Area Low Gray Level Emphasis
**Lung 2 fractions (2)**	**GLSZM** Low Gray Level Zone Emphasis	**GLSZM** Small Area Low Gray Level Emphasis	
**Lung 10 fractions (1)**		**GLSZM** Small Area Low Gray Level Emphasis	
**Lung 20 fractions (1)**	**GLSZM** Small Area Low Gray Level Emphasis		
**Lung 30 fractions (1)**	**GLSZM** Small Area Low Gray Level Emphasis		

ICCs were tested for consistency of dosiomic features across different numbers of fractions for all ROIs. The expectations of all dosiomic feature values were found to be consistent (ICC ≥ 0.9) with respect to different numbers of fractions (p-value < 0.01). Note that some dosiomic features that had ICC < 0.95 were GTV: “SmallAreaEmphasis” and “SizeZoneNonUniformityNormalized” from GLSZM, PTV: “ClusterProminence” from GLCM, Heart: SmallAreaLowGrayLevelEmphasis from GLSZM and Lung: SmallAreaLowGrayLevelEmphasis from GLSZM.

The results of percent differences (PDs) are shown in **Supplementary Figure S2**. We excluded patient number 14 from calculating the PD of the “Minimum” of the heart and lungs ROI due to the minimum dose being 0. Some of the dosiomic features had F_ori_ that differed from F_err_ by more than 50%. The dosiomic features that had a PD greater than 50% were “ClusterShade” (4 times) from GLCM, “SmallAreaEmphasis” (3 times), “SmallAreaHighGrayLevelEmphasis” (3 times), and “SmallAreaLowGrayLevelEmphasis” (3 times) from GLSZM.

## Discussion

Many studies have been performed on the stability of radiomic features, whereas only a few studies have reported on the stability of dosiomic features (6, 7, 22). This is the first report to introduce a concern about the stability of dosiomic features under interfractional error in radiation therapy.

Many ML and DL applications with texture analysis in the field of radiotherapy have been recently proposed (1, 3, 28–30). Many studies have extracted features from treatment plan dose distributions and used these features as input to machine learning models; however, such dose distributions might not represent the true dose distribution the patient received due to interfractional error. The effect of interfractional error is known to impact the equivalent uniform dose (EUD), the tumor control probability (TCP), and the normal tissue complication probability (NTCP) (13). Changes in EUD, TCP, and NTCP can affect the decision of the physician during treatment planning. In the same way, errors in dosiomic features can result in prediction errors in ML models. Therefore, this study aimed to investigate the impact of interfractional error on dosiomic features.

We calculated CVs from each dosiomic feature and compared them to assess stability. Most of the features in all total numbers of fractions (F2_err_, F10_err_, F20_err,_ and F30_err_) groups and all ROIs had high stability (very small CV and small CV). Comparing four ROIs, the lungs were the regions with high overall stability, while GTVs had low overall stability (**Figure 4**, **Supplementary Figure S1**). The most common dosiomic features with high variation compared to other features were similar to previous studies on dosiomic feature stability. For stability under different dose cube pixel spacings with a CV threshold of 0.3 (CV > 0.3), the stability of “Skewness” from the first-order statistic, “ClusterShade” and “ClusterProminence” from GLCM, and “SmallAreaLowGrayLevelEmphasis” from GLSZM were similar to our results (7). For the stability under dose grid resolution and dose calculation algorithms with a CV threshold of 0.5 (CV > 0.5), the stability of “Skewness” from the first-order statistic, “ClusterShade” from GLCM, and “SmallAreaLowGrayLevelEmphasis” from GLSZM were similar to our results (6). It should be noted that we only investigated the stability of dosiomic in primary lung cancer patients under interfractional error, while the previous studies have explored stability under other factors such as dose cube pixel spacing (7), dose grid resolution, and dose calculation algorithms (6) across several primary cancer such as breast and brain. Further study on investigating and comparing the results obtained with respect to other primary cancers should be listed as our future work.

CV also revealed that the stability of dosiomic features decreased as the total number of fractions decreased (**Figure 4** **)**. The high variation in the low total number of fractions group could be explained by the law of large numbers, causing a lower total number of fractions to have higher variance than a higher total number of fractions.

The ICC showed that some dosiomic features (**Table 1**) did not demonstrate excellent reproducibility defined by an ICC threshold of ≥ 0.9 (26, 27). A guideline of ICC by Terry K. Koo et al. (25) suggested that ICC values less than 0.5 could be defined as poor reproducibility, which corresponded to the dosiomic feature “SmallAreaLowGrayLevelEmphasis” from GLSZM extracted from the heart ROIs. ICC values of 0.5 ≤ ICC < 0.75 could be defined as moderate reliability, which corresponded to dosiomic features: “SmallAreaLowGrayLevelEmphasis,” “SmallAreaHighGrayLevelEmphasis,’’ “SmallAreaEmphasis” “SizeZoneNonUniformityNormalized” from GLSZM and “ClusterProminence” from GLCM (**Table 1**, **Supplementary Figure S3**). ICC values of 0.75 ≤ ICC < 0.90 and ICC ≥ 0.9 indicated good and excellent reproducibility. Using dosiomic features with poor or moderate reproducibility might result in limited generalizability in some ROIs while lowering the number of fractions might lower the reproducibility.

We reviewed the predictive dosiomic features reported in other studies. The results presented here were similar to those (31), which showed that the predictive features of treatment response had high stability (32). “LongRunHighGrayLevelEmphasis” from GLRLM “Contrast” from GLCM and “LowGrayLevelEmphasis” from GLDM were selected as predictive factors in genitourinary and gastrointestinal complications in prostate cancer (1), radiation pneumonitis in lung cancer (3), and locoregional recurrences in head and neck cancer (30), respectively. These selected features had high stability (CV < 10%) and excellent reproducibility (ICC ≥ 0.9) according to the number of delivered fractions.

We investigated the PD between F_ori_ and F_err_, comparing four ROIs. The lungs were the regions that had the lowest overall PD, and GTVs were the regions with the highest overall PD (**Supplementary Figure S2**). Note that a small or large PD between F_ori_ and F_err_ does not always lead to small or large errors in the predictive performance of a model, as there are many other factors, such as model parameters and techniques, in developing the model that may impact performance. For example, if the dosiomic features with large errors are normalized to small values and the weight of the features is small, then the change in the model result will also be small. The features are generally normalized before being input to the model, and models usually have regularization constraints.

A limitation of this study was that our data only included lung cancer patients. Different cancer types or different ROIs may give different feature values. However, from our results, the value of CVs and ICC in four tested ROIs showed a similar pattern (although the magnitudes were not the same). For example, in the same ROI, some features in GLSZM showed high CVs (**Supplementary Figure S1**) or high 1-ICC (**Supplementary Figure S3**) when compared with other dosiomic features. The difference in magnitude of stability may arise from the total dose in that region. Therefore, we also expected a similar pattern of stability in different cancer sites, with the magnitude of stability depending on the total dose. The dose to the heart ROIs was lower than the dose to the lung ROIs (**Supplementary Table S3**). However, lung ROIs had higher overall stability than heart ROIs. We believe that this came from the interfractional error, which caused the dose to the heart ROIs to be different in each dose distribution (**Supplementary Figure S4** **)**, while the dose to the lungs was similar even with interfractional error.

In summary, this study investigated the stability of dosiomic features with IMRT and VMAT in a lung cancer dataset. Our results showed that some dosiomic features might not be reliable under interfractional error and with lower fraction numbers, even more susceptible to the effects of interfractional error resulting in unstable features. Our study also investigated plans with a higher dose per fraction and lower number of fractions than usual of IMRT/VMAT plans. Therefore, we expected that the treatment plan using the stereotactic body radiotherapy (SBRT) technique would yield similar results as the number of fractions 2 and 10. The stability of texture features should be further investigated using SBRT datasets.

## Data Availability Statement

The datasets presented in this article are not readily available. Requests to access the datasets should be directed to yodchanan.won@mahidol.ac.th.

## Ethics Statement

The studies involving human participants were reviewed and approved by the Human research ethics committee, Faculty of Medicine Ramathibodi Hospital, Mahidol University (IRB MURA2021/283).

## Author Contributions

CP, NS, and NT contributed to the acquisition of the data. CP, PP, NS, and SK contributed to the experimental design and statistical analysis. CP contributed to processing the data and feature extraction. CP and NS drafted the manuscript. SK, PP, and YW were senior authors supervising the project. All authors read and approved the final manuscript.

## Funding

This project is supported in part by the National Higher Education Science Research and Innovation Policy Council, PMU B (B05F640079).

## Conflict of Interest

The authors declare that the research was conducted in the absence of any commercial or financial relationships that could be construed as a potential conflict of interest.

## Publisher’s Note

All claims expressed in this article are solely those of the authors and do not necessarily represent those of their affiliated organizations, or those of the publisher, the editors and the reviewers. Any product that may be evaluated in this article, or claim that may be made by its manufacturer, is not guaranteed or endorsed by the publisher.
