# Supplementary material for: Impact of Interfractional Error on Dosiomic Features
Source: Front Oncol. 2022 Jun 10;12:726896. doi: 10.3389/fonc.2022.726896 (PMC9231355; doi:10.3389/fonc.2022.726896)
Supplement: Supplementary file 1 [file DataSheet_1.docx]

***Supplementary Material***

# Supplementary Table

**Supplementary Table 1:** Clinical characteristics of patients in this study.

| Patient | TNM staging | Primary tumor location | Age | Sex (M/F) | COPD |
| --- | --- | --- | --- | --- | --- |
| 1 | T4N3M0 | LUL | 62 | M | No |
| 2 | T2aN1M0 | RLL | 68 | F | Yes |
| 3 | T2bN3M0 | LUL | 65 | F | No |
| 4 | T2bN2M0 | RLL | 66 | M | No |
| 5 | T3N3M0 | RUL | 48 | F | No |
| 6 | T2bN0M0 | LUL | 74 | M | No |
| 7 | T4N3M0 | LUL | 65 | M | Yes |
| 8 | T3N3M0 | RUL | 63 | M | No |
| 9 | T1N3M0 | RUL | 70 | F | No |
| 10 | T4N2M0 | RUL | 79 | M | No |
| 11 | T3N3M0 | LUL | 58 | M | No |
| 12 | T1bN2M0 | LLL | 60 | F | No |
| 13 | T4N3M0 | LUL | 73 | M | Yes |
| 14 | T4N3M0 | LUL | 59 | M | No |
| 15 | T1N2M0 | RUL | 74 | F | Yes |

**Supplementary Table 2:** Radiation setting of patients in this study.

| Patient | Total prescription dose (Gy) | Prescription dose per fraction (Gy) | Radiation technique | CT image | Normalization |
| --- | --- | --- | --- | --- | --- |
| 1 | 60 | 2 | VMAT | Non-contrast | 97% |
| 2 | 60 | 2 | VMAT | 4D average | 97.50% |
| 3 | 60 | 2 | VMAT | 4D average | 100% |
| 4 | 50.4 | 1.8 | VMAT | Non-contrast | 99.20% |
| 5 | 60 | 2 | VMAT | 4D average | 98% |
| 6 | 62 | 2 | VMAT | 4D average | 100% |
| 7 | 66 | 2 | VMAT | 4D average | 98.50% |
| 8 | 59.4 | 1.8 | IMRT | Non-contrast | 95.50% |
| 9 | 59.4 | 1.8 | VMAT | Non-contrast | 100% |
| 10 | 60 | 2 | VMAT | Non-contrast | 100% |
| 11 | 54 | 1.8 | IMRT | Non-contrast | 97% |
| 12 | 59.4 | 1.8 | IMRT | Non-contrast | 98.50% |
| 13 | 60 | 2 | VMAT | 4D average | 100% |
| 14 | 60 | 2 | IMRT | Non-contrast | 96.50% |
| 15 | 59.4 | 1.8 | IMRT | Non-contrast | 100% |

**Supplementary Table 3:** Dose profile of all ROIs.

| Patient | GTV mean dose  (min-max dose) | PTV mean dose  (min-max dose) | Heart mean dose  (min-max dose) | Lung mean dose  (min-max dose) |
| --- | --- | --- | --- | --- |
| 1 | 62.81 (43.83-67.81) | 60.86 (37.02-67.81) | 9.79 (0.4-61.22) | 17.79 (0.43-65.84) |
| 2 | 62.75 (53.93-65.69) | 61.34 (25.41-65.71) | 18.9 (1.54-56.15) | 16.87 (0.45-65.69) |
| 3 | 63.57 (60.75-65.62) | 62.89 (55.37-66.33) | 2.15 (0.17-37.83) | 13.78 (0.3-65.47) |
| 4 | 53.74 (50.93-55.98) | 53.4 (47.1-55.98) | 12.24 (2.96-54.5) | 14.46 (0.91-55.78) |
| 5 | 62.66 (60.55-64.05) | 62.39 (56.26-65.49) | 1.78 (0.16-60.96) | 12.59 (0.13-65.29) |
| 6 | 64.11 (62.06-66.13) | 64.02 (61.39-66.13) | 4.37 (0.38-62.1) | 13.41 (0.23-66.08) |
| 7 | 67.98 (64.35-72.16) | 67.84 (63.71-72.16) | 3.0 (0.32-50.09) | 14.66 (0.05-70.45) |
| 8 | 61.91 (59.73-63.93) | 60.64 (26.08-64.87) | 10.11 (0.41-64.87) | 18.7 (0.55-63.95) |
| 9 | 62.46 (60.83-63.38) | 61.52 (56.54-63.7) | 4.97 (0.18-63.7) | 14.85 (0.2-63.22) |
| 10 | 62.39 (58.8-65.81) | 61.65 (32.04-66.84) | 1.38 (0.29-5.15) | 13.84 (0.23-65.22) |
| 11 | 55.78 (54.31-57.92) | 55.35 (42.59-58.85) | 11.39 (0.51-58.26) | 16.32 (0.43-59.18) |
| 12 | 61.22 (59.43-62.96) | 60.72 (55.7-64.11) | 18.26 (0.81-64.32) | 12.64 (0.27-63.52) |
| 13 | 62.35 (55.73-66.56) | 61.2 (46.11-66.56) | 6.28 (0.36-62.17) | 11.98 (0.23-64.86) |
| 14 | 61.85 (60.41-63.22) | 61.81 (43.12-64.14) | 0.49 (0.0-1.59) | 5.29 (0.0-63.15) |
| 15 | 60.61 (57.2-62.55) | 57.05 (44.82-63.3) | 5.8 (0.27-62.4) | 15.63 (0.26-61.59) |

**Supplementary Table 4:** All features used in this study.

|  | Features |
| --- | --- |
| First Order  (18 features) | 10thPercentile, 90thPercentile, Energy, Entropy, InterquartileRange, Kurtosis, Maximum, AbsoluteDeviation, Mean, Median, Minimum, Range, RobustMeanAbsoluteDeviation, RootMeanSquared, Skewness, TotalEnergy, Uniformity, Variance |
| Gray Level Co-occurrence Matrix (GLCM)(24 features) | Autocorrelation, JointAverage, ClusterProminence, ClusterShade, ClusterTendency, Contrast, Correlation, DifferenceAverage, DifferenceEntropy, DifferenceVariance, JointEnergy, JointEntropy, InformationalMeasureOfCorrelation (IMC), InformationalMeasureOfCorrelation2 (IMC2), InverseDifferenceMoment (IDM), MaximalCorrelationCoefficient (MCC), InverseDifferenceMomentNormalized (IDMN), InverseDifference (ID), InverseDifferenceNormalized (IDN), InverseVariance, MaximumProbability, SumAverage, SumEntropy, SumOfSquares |
| Gray Level Run Length Matrix (GLRLM)(16 features) | ShortRunEmphasis, LongRunEmphasis, GrayLevelNonUniformity, GrayLevelNonUniformityNormalized, RunLengthNonUniformity, RunLengthNonUniformityNormalized, RunPercentage, GrayLevelVariance, RunVariance, RunEntropy, LowGrayLevelRunEmphasis, HighGrayLevelRunEmphasis, ShortRunLowGrayLevelEmphasis, ShortRunHighGrayLevelEmphasis, LongRunLowGrayLevelEmphasis, LongRunHighGrayLevelEmphasis |
| Gray Level Size Zone Matrix (GLSZM)(16 features) | SmallAreaEmphasis, LargeAreaEmphasis, GrayLevelNonUniformity, GrayLevelNonUniformityNormalized, SizeZoneNonUniformity, SizeZoneNonUniformityNormalized, ZonePercentage, GrayLevelVariance, ZoneVariance, ZoneEntropy, LowGrayLevelZoneEmphasis, HighGrayLevelZoneEmphasis, SmallAreaLowGrayLevelEmphasis, SmallAreaHighGrayLevelEmphasis, LargeAreaLowGrayLevelEmphasis, LargeAreaHighGrayLevelEmphasis |
| Neighbouring Gray Tone Difference Matrix (NGTDM)(5 features) | Coarseness, Contrast, Busyness, Complexity, Strength |
| Gray Level Dependence Matrix (GLDM)(14 features) | SmallDependenceEmphasis, LargeDependenceEmphasis, GrayLevelNonUniformity, DependenceNonUniformity, DependenceNonUniformityNormalized, GrayLevelVariance, DependenceVariance, DependenceEntropy, LowGrayLevelEmphasis, HighGrayLevelEmphasis, SmallDependenceLowGrayLevelEmphasis, SmallDependenceHighGrayLevelEmphasis, LargeDependenceLowGrayLevelEmphasis, LargeDependenceHighGrayLevelEmphasis |

# Supplementary Figures

| 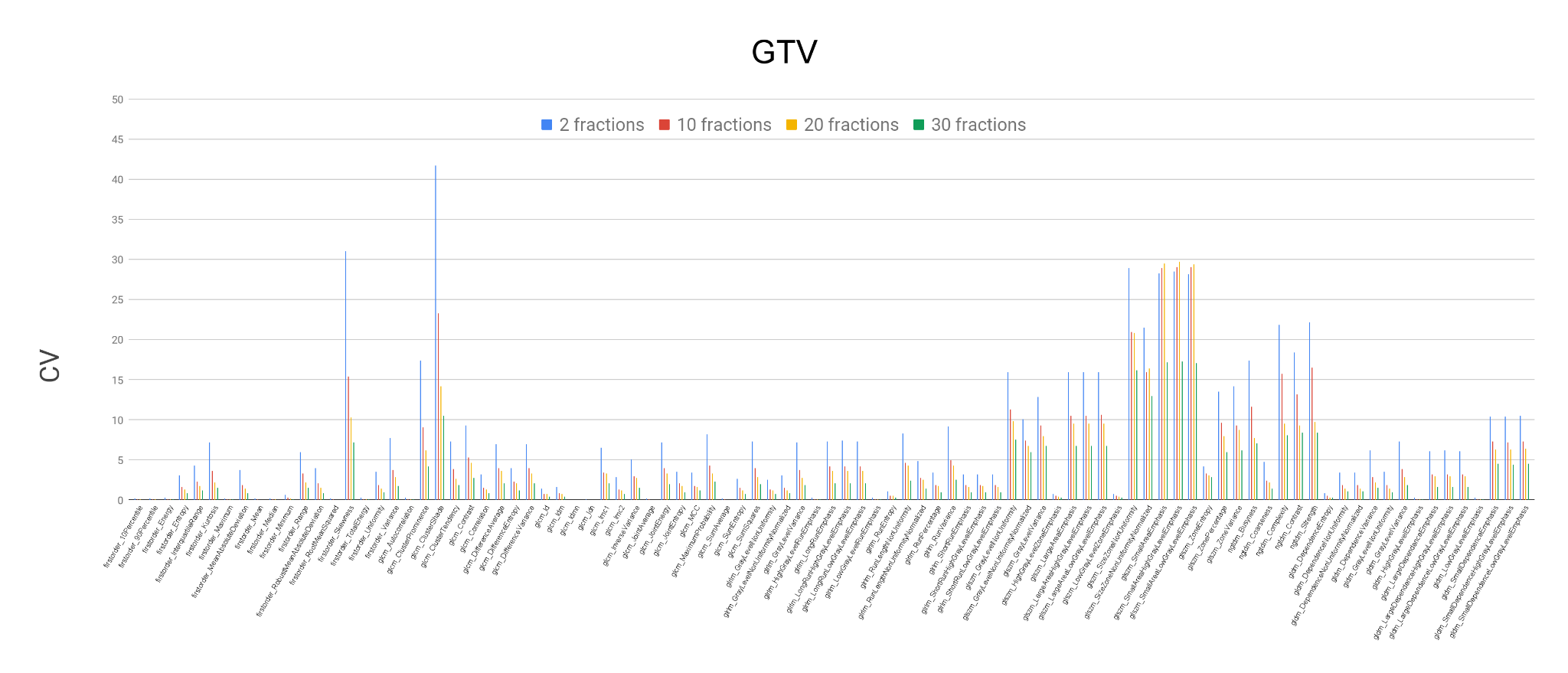 |
| --- |
| 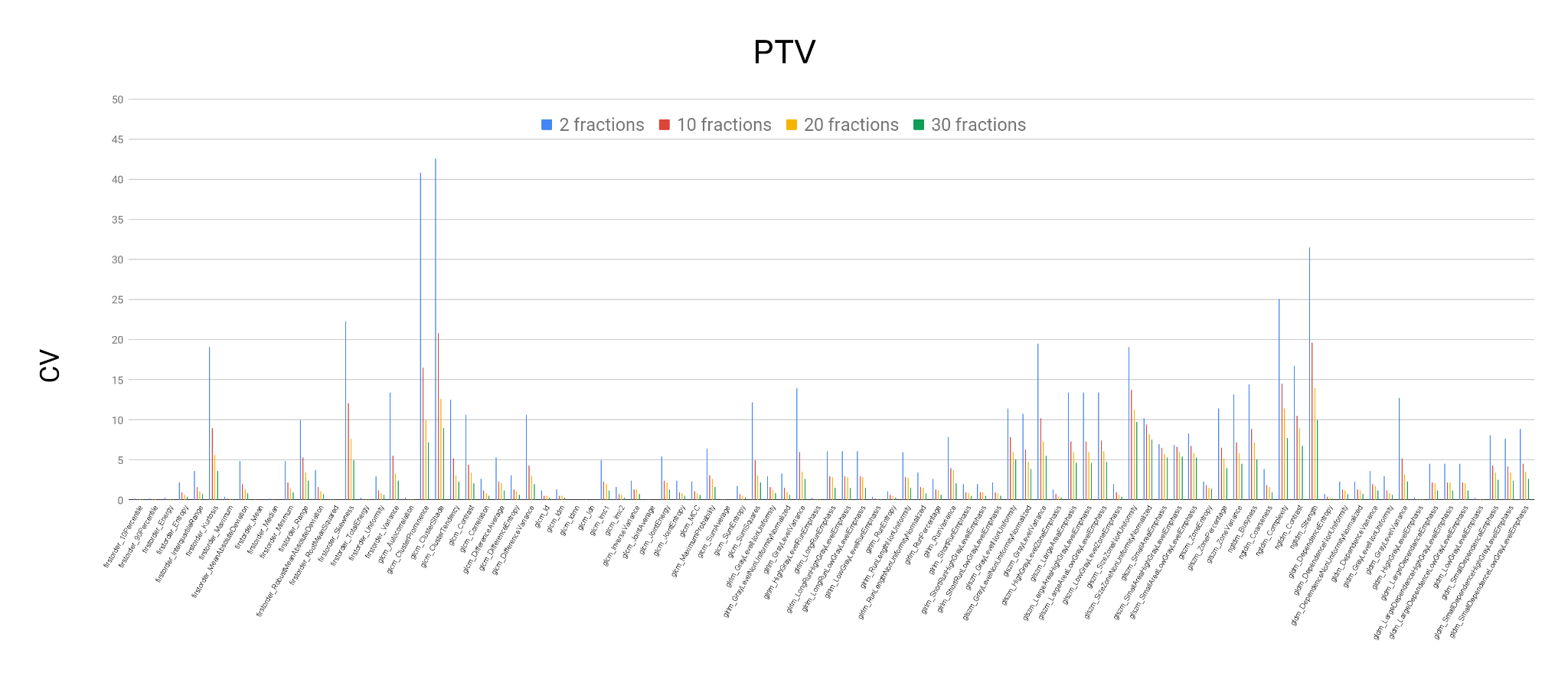 |
| 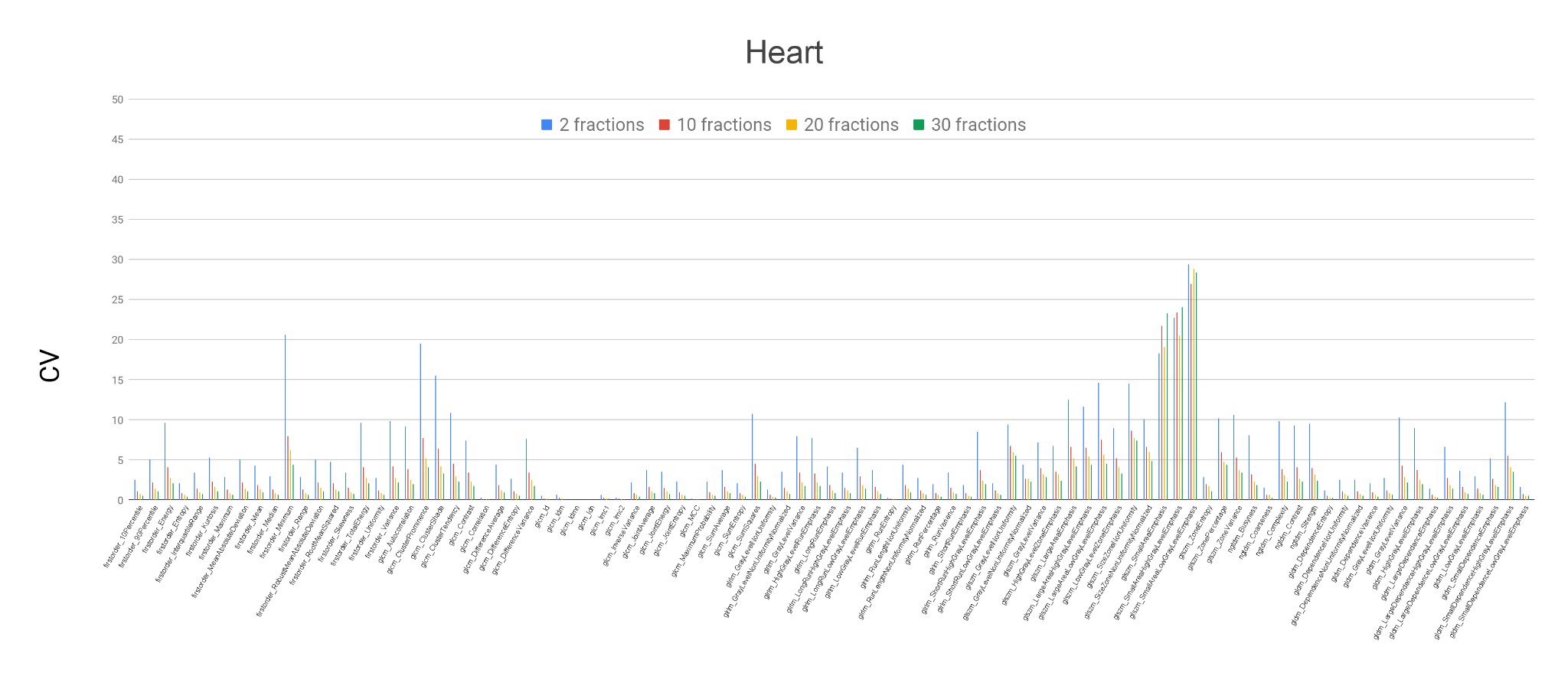 |
| 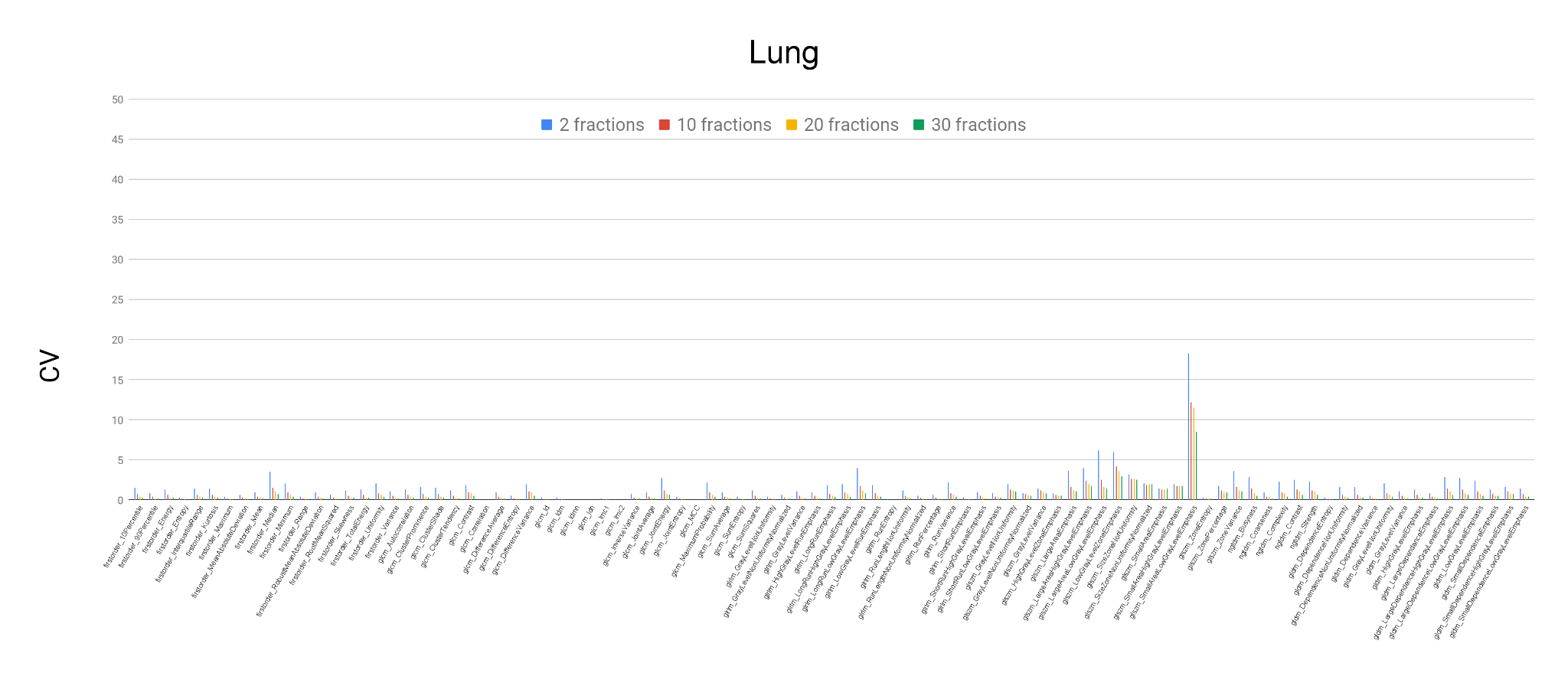 |

**Supplementary Figure 1:** CV was calculated for all dosiomic features for different ROIs and different number of fractions.

| 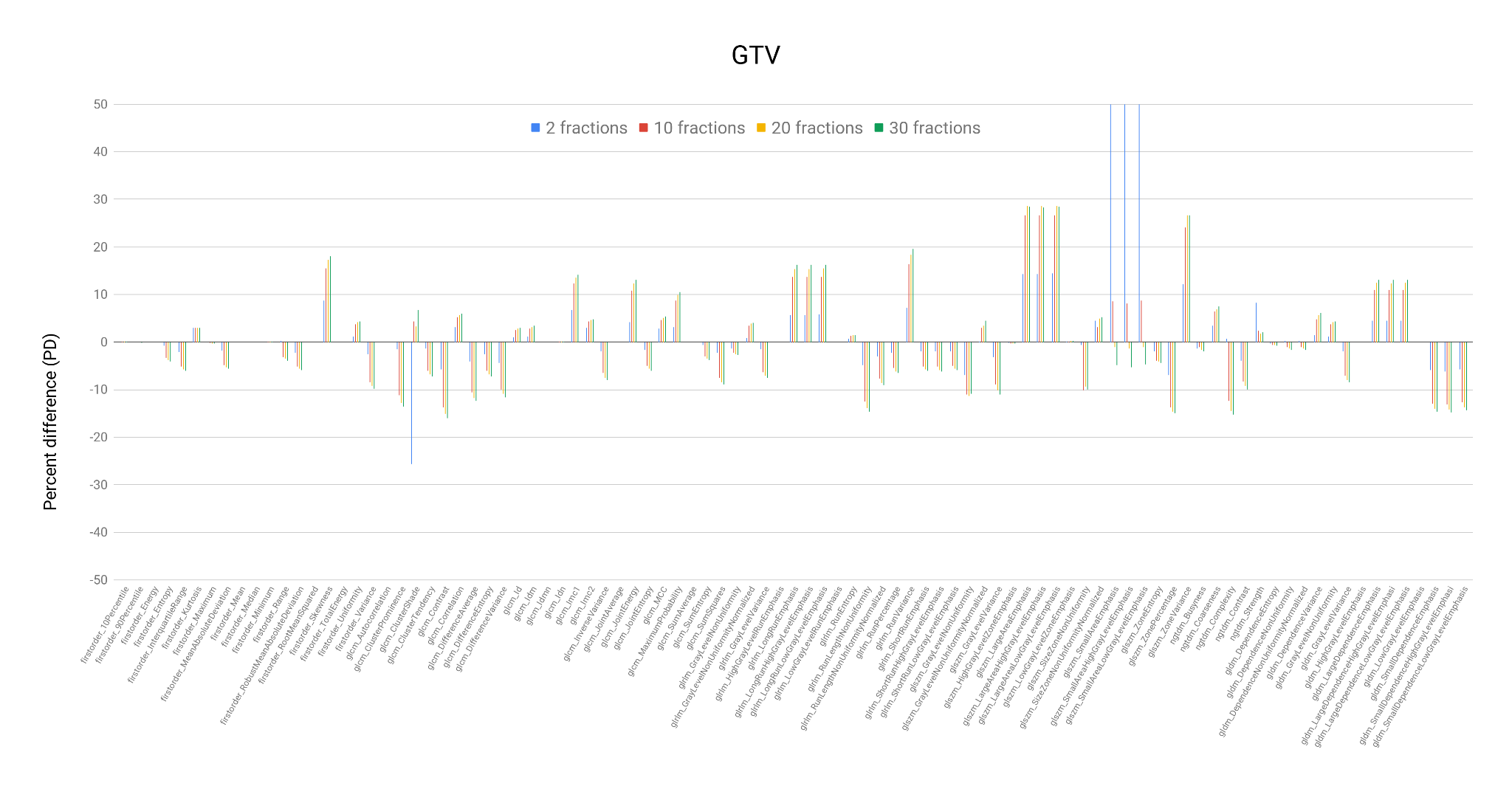 |
| --- |
| 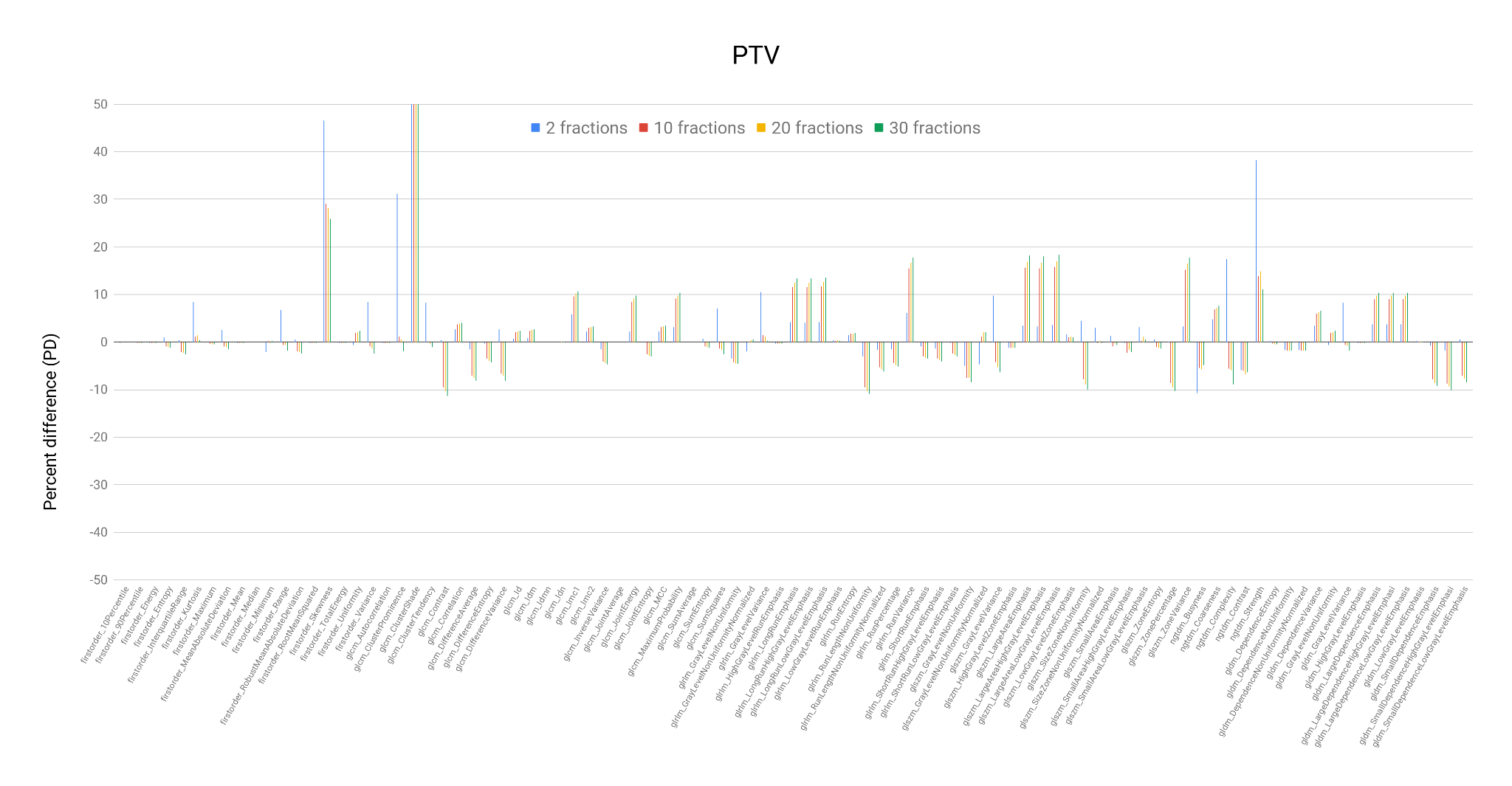 |
| 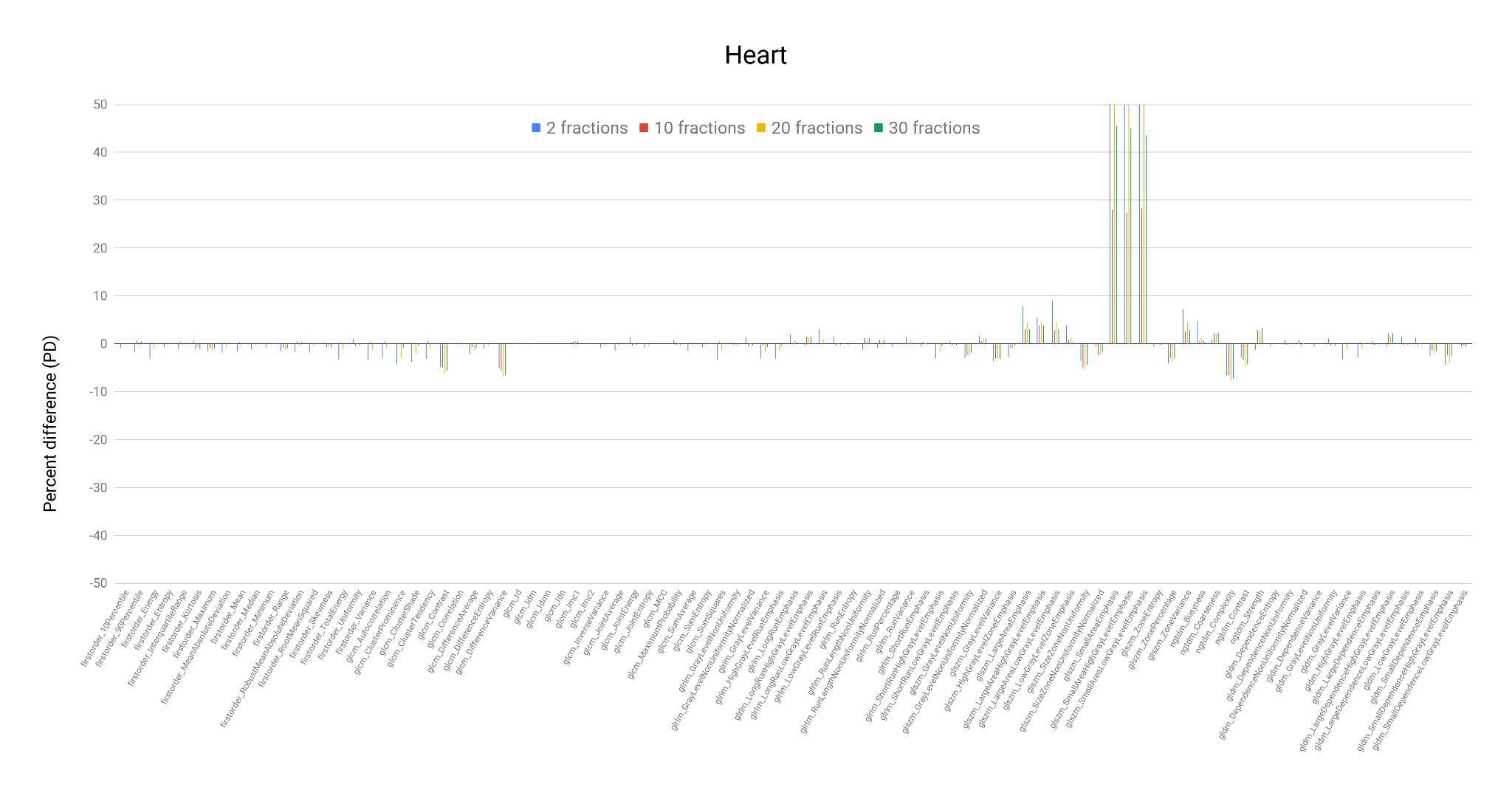 |
| 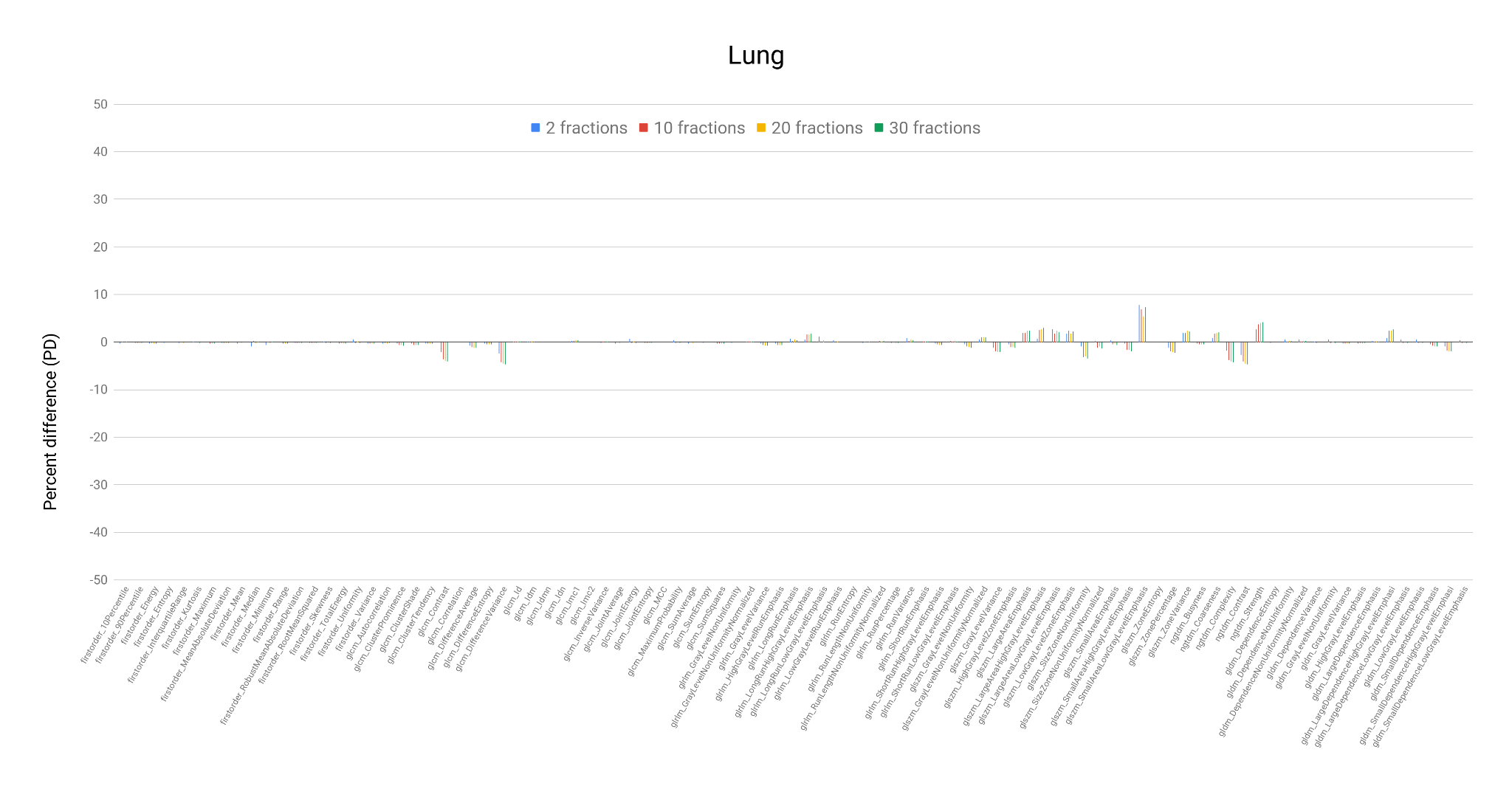 |

**Supplementary Figure 2:** PD was calculated for all dosiomic features for different ROIs and different number of fractions.

| 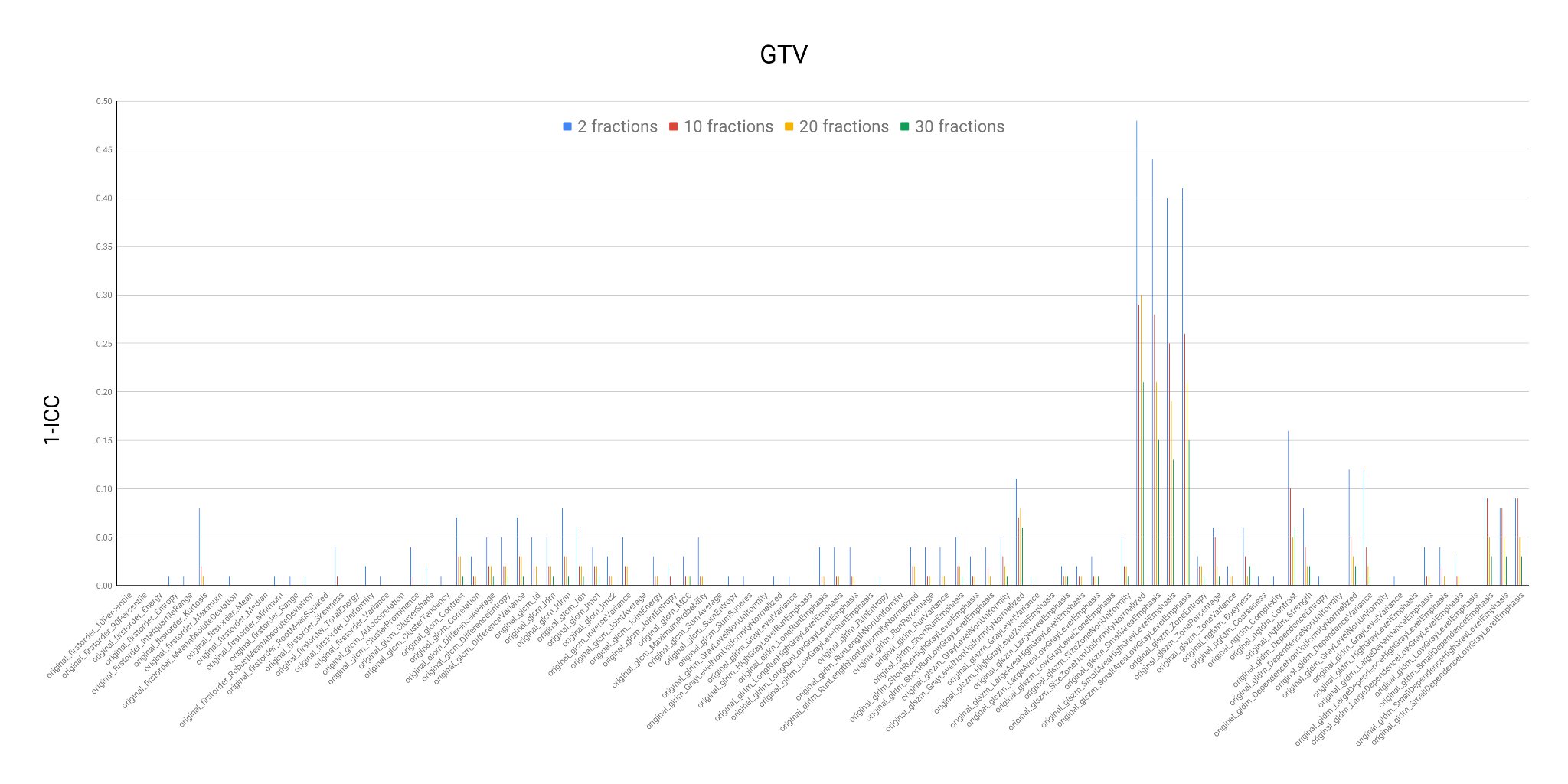 |
| --- |
| 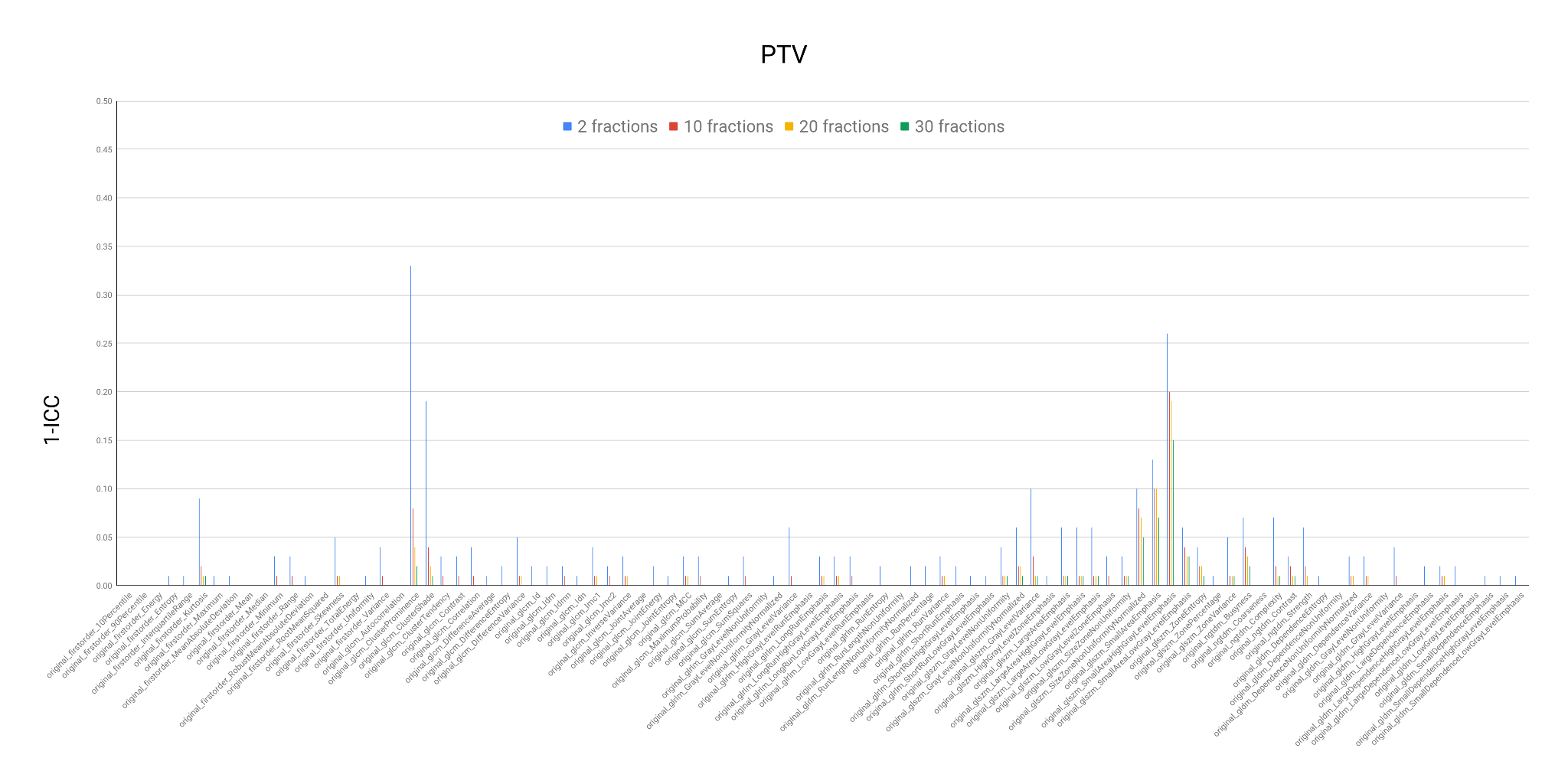 |
| 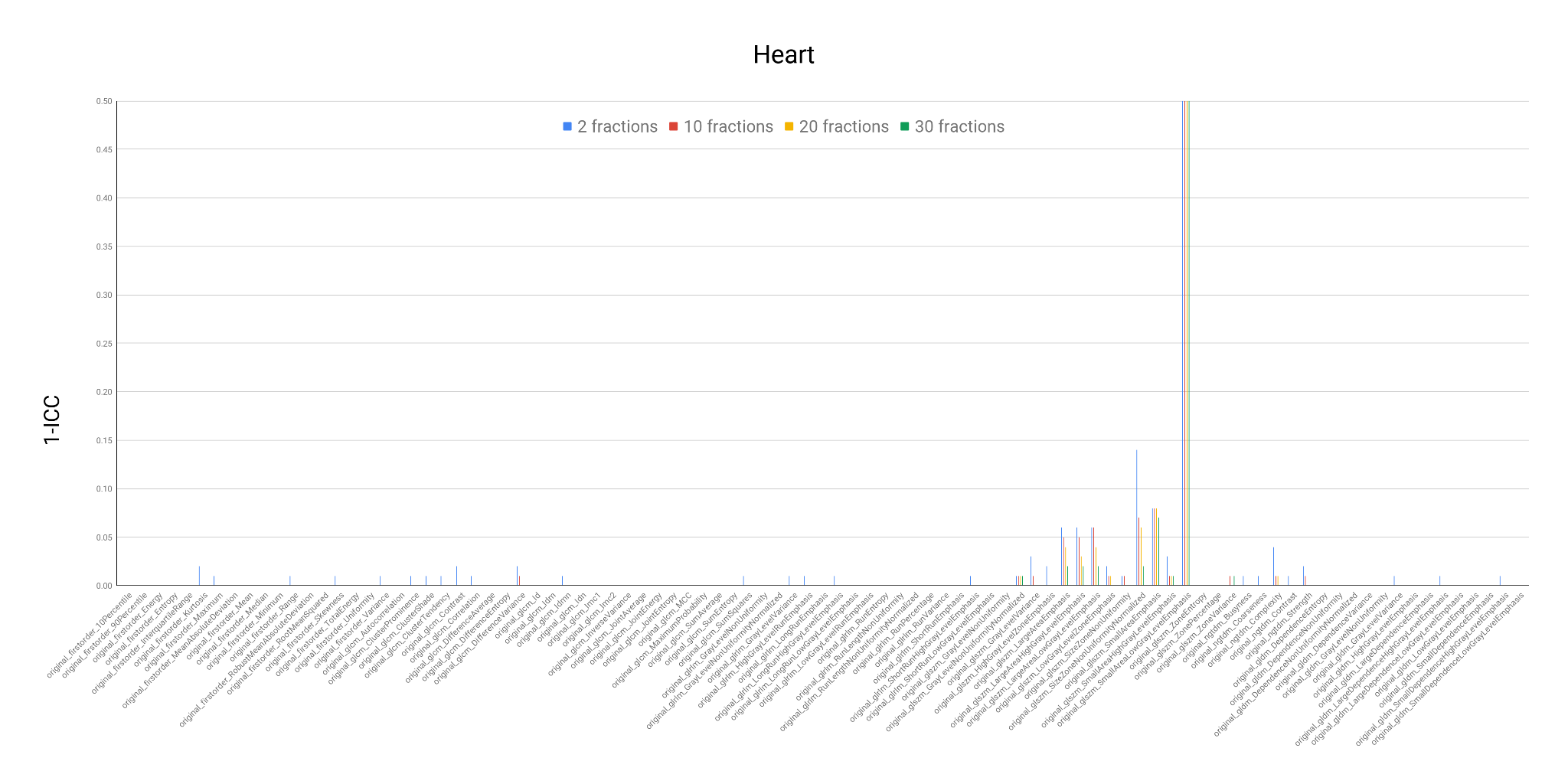 |
| 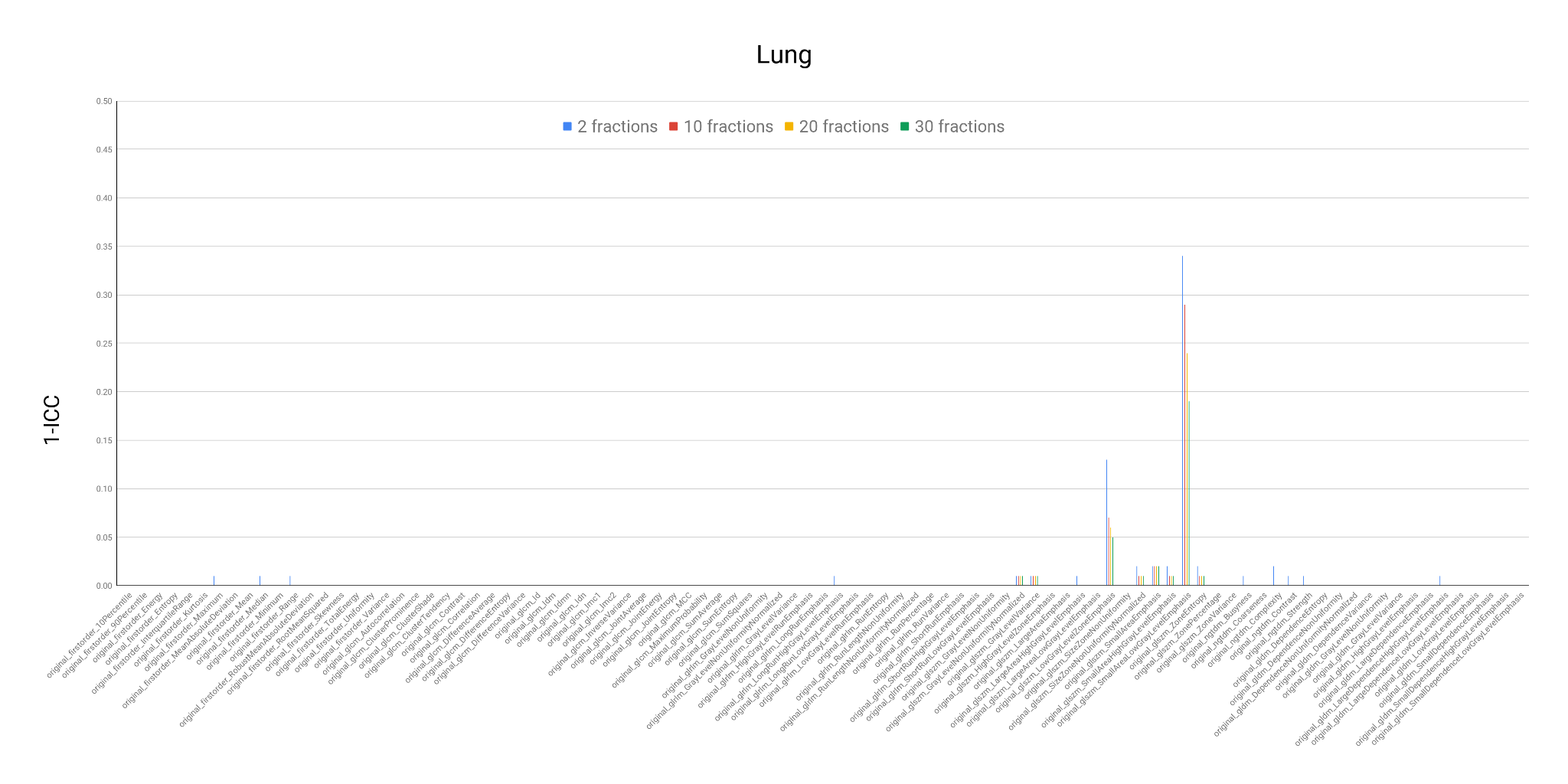 |

**Supplementary Figure 3:** 1-ICC was calculated for all dosiomic features for different ROIs and different number of fractions with p-value < 0.01.


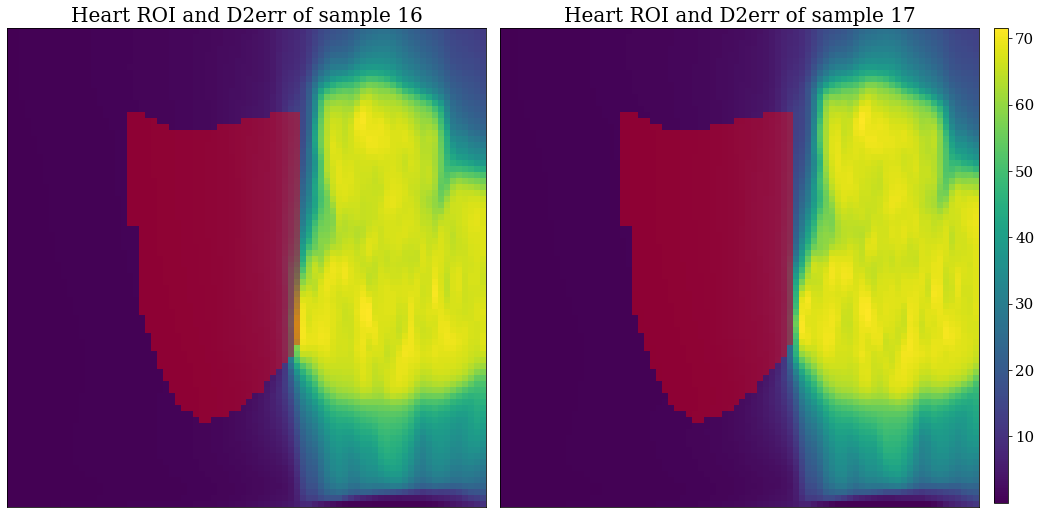


**Supplementary Figure 4:** Dose distribution with inter-fractional error of 2 fractions (D2_err_) of patient 14. Heart ROI on the left overlap with high dose area and small shift resulted in large dose variation.
